# The Recognition of Facial Expressions Under Surgical Masks: The Primacy of Anger

**DOI:** 10.3389/fnins.2022.864490

**Published:** 2022-06-16

**Authors:** Alice M. Proverbio, Alice Cerri

**Affiliations:** Cognitive Electrophysiology Laboratory, Department of Psychology, University of Milano-Bicocca, Milan, Italy

**Keywords:** emotions, face masking, facial expression, face processing, sex difference, empathy

## Abstract

**Background:**

The need to wear surgical masks in everyday life has drawn the attention of psychologists to the negative effects of face covering on social processing. A recent but not homogeneous literature has highlighted large costs in the ability to recognize emotions.

**Methods:**

Here it was investigated how mask covering impaired the recognition of facial mimicry in a large group of 220 undergraduate students. Sex differences in emotion recognition were also analyzed in two subgroups of 94 age-matched participants. Subjects were presented with 112 pictures displaying the faces of eight actors (4 women and 4 men) wearing or not wearing real facemasks, and expressing seven emotional states (neutrality, surprise, happiness, sadness, disgust, anger and fear). The task consisted in categorizing facial expressions while indicating the emotion recognizability with a 3-point Likert scale. Scores underwent repeated measures ANOVAs.

**Results:**

Overall, face masking reduced emotion recognition by 31%. All emotions were affected by mask covering except for anger. Face covering was most detrimental to sadness and disgust, both relying on mouth and nose expressiveness. Women showed a better performance for subtle expressions such as surprise and sadness, both in masked and natural conditions, and men for fear recognition (in natural but especially masked conditions).

**Conclusion:**

Anger display was unaffected by masking, also because corrugated forehead and frowning eyebrows were clearly exposed. Overall, facial masking seems to polarize non-verbal communication toward the happiness/anger dimension, while minimizing emotions that stimulate an empathic response in the observer.

## Introduction

It is known that surgical masks (used pervasively to counter the transmission of coronavirus) might negatively affect and impair social processing. Impairment might concern the recognition of face identity (Carragher and Hancock, 2010; Noyes et al., 2021), emotion reading (Roberson et al., 2012; Carbon, 2020; Ruba and Pollak, 2020; Calbi et al., 2021; Grundmann et al., 2021; Marini et al., 2021; Carbon et al., 2022), trustworthiness judgment (Biermann et al., 2021), face likability and closeness impression (Grundmann et al., 2021), as well as speech comprehension (Singh et al., 2021). Relatedly, previous literature showed that face blurring impairs the understanding of emotional signals including body language (Proverbio et al., 2018). Although emotions conveyed by bodily expressions are quite easily recognizable (de Gelder et al., 2015), face obscuration reduces pantomime comprehension in healthy subjects, as opposed to patients with bilateral amygdala damage (Adolphs et al., 2003). This indicates how facial mimicry is crucial in nonverbal communication. For example, when facial expressions are incongruent with bodily expressions (of anger, for instance) response times are much slower during a matching-to-sample task in controls (Kret and de Gelder, 2013), thus suggesting that bodily expressions are better recognized when accompanied by a face that expresses the same emotion (Meeren et al., 2005).

To investigate at which extent face covering impaired social communication Grundmann et al. (2021) performed a large study on 191 individuals of different ages and sexes and found that facemasks diminished people’s ability to accurately categorize facial expressions and affected the perceptions of person trustworthiness, likability, and closeness.

Generally, the mouth region is thought to be most informative for happy, surprised and disgusted expressions, while the eyes area is considered more informative for fearful and angry expressions. For example, the white sclera expansion, typical of fear display, is especially at the basis of its innate recognition (e.g., Jessen and Grossmann, 2014; Barrett, 2018). Both the mouth and eyes areas are informative for neutral and sad expressions (Smith et al., 2005;Wegrzyn et al., 2017). In addition, joy can very well detected through the smiling mouth, but especially the “smiling” eyes. Years of psychological (e.g., Ekman et al., 1998) and lately engineering research on pattern recognition (e.g., Ugail and Al-dahoud, 2019) have shown that the more reliable indicators of a genuine happy facial expression are indeed the eyes. Angry facial expressions are associated with a strong activation of the corrugator supercilii (i.e., the muscle involved in frowning), whereas happy facial expressions are associated with a strong activation of the zygomaticus major, (the muscle involved in smiling) (Rymarczyk et al., 2019). Based on the above findings we expected lower costs in accuracy due to the covering of the lower part of the face (face masking) during recognition of anger and happiness expressions, but the available literature was not completely homogeneous at this regard.

Noyes et al. (2021) recently explored the effect of masks and sunglasses wearing on familiar and unfamiliar face matching and emotion categorization in 100 participants. They found that, while masks did not reduce the recognition of angry faces, facial expressions of disgust, happy, fear and surprise were most affected by it. A large reduction in categorization accuracy for disgust expressions was found in particular. Sadness detection was difficult both mask less and with mask covering, so that the performance was not significantly impaired by masking. Results are not fully consistent across studies. In a recent study by Marini et al. (2021) investigating the impact of facemasks on emotion recognition (but only with three emotions) they showed an impaired recognition of sad and fearful expressions in the masked condition, with no effect on neutral expressions. Among the three expressions, sadness was the most affected and happiness the least affected. In this study, sadness was more hardly detected with mask covering the mouth area.

One of the problems with the available studies is that many of them digitally applied a mask or a foulard on the face picture in order to create identically expressive faces, across the masked vs. non-masked category (e.g., Carragher and Hancock, 2010; Carbon, 2020; Calbi et al., 2021; Grundmann et al., 2021; Marini et al., 2021; Carbon et al., 2022). While this procedure might assure an optimal matching between masked and unmasked expressions, however it lacks likelihood and ecological value. Indeed, digitally applied masks are not stretched by the facial expression thus reducing the verisimilitude. Furthermore, they deprive the visual image of details that are present in the real masked face, such as mask sucking or folding. In reality, surgical masks are, for example, deformed by the vertical opening of the mouth in expressions like surprise or laughing, or during verbal speech; likewise, they are stretched horizontally for smiling. Indeed the masks adapt to, and reveal, the underneath muscular movements, which can be picked up by an observer. In order to maintain the visibility of mask bending and stretching due to underneath facial mimicry, in this study, actors wore real surgical masks during shooting. Several repetitions and much effort was devoted to the perfect matching between expressions produced with or without masks.

The aim of the study was to gain clear knowledge on the effects of face masking on the comprehensibility of a large variety of facial expressions (i.e., the six basic Ekman emotions: fear, anger, joy, sadness, disgust, and surprise plus neutrality) by using real and non-digital facemasks, unlike many of the previous studies quoted above. In fact, it is possible that digital masks further limited the possibility of recognizing facial mimicry because they are fixed and do not show dynamic deformations of the fabric, made possible by its elasticity. For example, real masks can show inhalation-related sucking associated with startle reaction in the surprise or fear expressions. Again, they can also show vertical and horizontal stretching of the tissue due to smiling or nose wrinkling. Therefore, it is possible that emotion recognition under digital facemasks was currently under-estimated.

In addition, we wished to investigate if face masking affected the two sexes differently. According to the available psychological and neuroscientific literature, overall, females would be more accurate in identifying emotional facial expressions then males (e.g., McClure, 2000; Montagne et al., 2005; Proverbio et al., 2006; Proverbio, 2021). Indeed, a recent study involving perception of masked faces (Grundmann et al., 2021) showed that being a man was associated with a reduced accuracy in emotion recognition than being a woman, without specific interactions with face masking conditions.

## Materials and Methods

### Participants

220 undergraduate students of local University self-recruited through online advertisement posted on the student’s web site. Six of them were excluded because older than 35 years. They aged between 18 and 35 years (mean = 21.617, SD = 2.91) and 47 of them were males). Experiments were conducted with the understanding and written consent of each participant according to the Declaration of Helsinki (BMJ 1991; 302: 1194) with approval of the Ethical Committee of the Psychology department of local University approved the study (protocol number: RM-2021-401). It was conducted online from June 25 until July 8 2021 and programmed in Google forms https://www.google.com/forms. Participation was free and not rewarded.

### Stimuli

**FIGURE 1:**
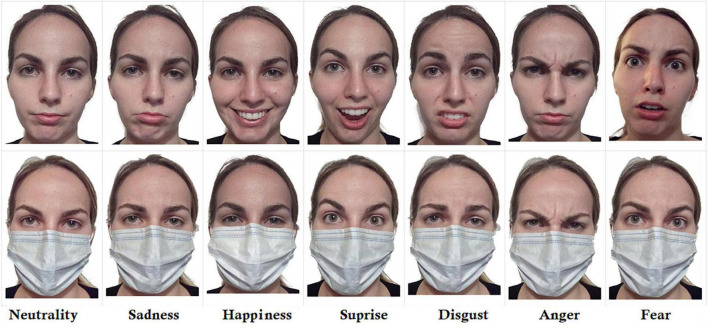
Example of stimuli (facial expressions) in the natural and masked condition. Overall, stimuli were created by taking photographs of natural expressions of eight actors (four male and four females) concentrated on their inner imaginary emotional state, through the Stanislavsky method. Masks were worn in reality and not digitally recreated. This revealed, for example the mouth/lips contraction associated with anger, the large mouth opening associated with disgust, the air intake (inhalation) characteristic of surprise or fear resulting in evident mask sucking.

10 actors (master psychology students) of Caucasian ethnicity were recruited (five females and five males) aging 23 years on average (SD = 1.333) for photos taking. High-resolution pictures of their faces were self- taken with a cell phone at about 40 cm of distance in light controlled conditions, while standing up against a white wall. Actors were required to avoid wearing earrings, glasses, make up, hairpins, pliers, any type of hair embellishments, mustaches, beard. They were also instructed to wear a black t-shirt and gather the hair behind the head. The pictures of two actors were discarded in that showing a different mimicry in the natural vs. masked condition; their pictures were therefore used only as stimuli for the training phase, to accustom the subjects to the task, without showing them the faces selected for the experimental phase. For each of the seven emotions, actors were instructed to imagine a vivid emotional state, while concentrating on a specific autobiographic scenario through the Stanislavsky method, and express it spontaneously while ignoring the presence/lack of surgical masks. For “surprise” emotion, they were instructed to think of a positive surprise. They trained repeatedly in order to reach the same degree of intensity across subjects and emotions (see Figure 1 for some examples). Each of the 10 actors provided written consent and filled in the privacy release form.

Stimulus set was validated on a group of 50 students (25 females, 24 males and 1 gender fluid) aging on average 23.7 years (min = 17, max = 34 years). All participants had normal vision, no neurological or psychiatric deficits and possessed diploma, BA or Master degrees. Participants were shown, randomly mixed and once at a time, the 56 pictures relative to the seven facial expressions acted by the eight female and male actors. Subjects were required to rapidly observe the picture and decide which one of the seven emotions typed below was more appropriate to describe the viewed facial expression, by clicking a check mark within a few seconds. Pictures were displayed at the center of the screen and the experimental session lasted 10 min.

Overall performance for correctly identifying facial emotions in unmasked faces was remarkably high = 87.35% (with a chance rate of 16.7%). No participant performed below an overall rate of 75.0%. In more details, accuracy was 98.47% for joy, 86.73% for surprise, 80.1% for sadness, 89.29% for anger, 72.70% for fear, 85.97% for disgust and 98.21% for neutrality. These recognition rates (in line with the data reported by Carbon, 2020; Carbon et al., 2022) outperform the accuracy of recognizing facial expressions reported by other studies in the literature (e.g., 57.85% for anger and disgust in Aviezer et al. (2008) and 57.85 for negative emotions in Derntl et al. (2009) thus supporting the qualitative validity of the stimuli.

Stimulus set was also evaluated for facial attractiveness by a further group of 12 students (seven females and five males) aged between 18 and 25 years. Judges were requested to evaluate the attractiveness of neutral unmasked pictures of all identities, by using a 3-point Likert scale, where 1 stood for “not attractive,” 2 for “average” and 3 for “attractive.” The results showed a perfect balance across the two sexes and indicated an “average” degree of attractiveness for the facial stimuli (Females = 1.83; SD = 0.78; Males = 1.82; SD = 0.76). This characteristic of stimuli promotes the generalizability of results to the normally looking population

### Procedure

After giving written and informed consent participants were administered a questionnaire about demographic information (such as age, sex, manual dexterity, educational qualification and e-mail address). This section was followed by the emotion-recognition task, consisting in 112 experimental trials, in which participants were first shown a portrait photograph of an adult face to be inspected for about 2 s. The images were equiluminant as assessed by subjecting their luminance values to an analysis of variance (*F* = 0.099, *p* = 0.992). Photos were in color, had the same size (3.37 cm × 5 cm; 199 × 295 pixels; 3° 22′ × 5°) and were displayed at the center of the screen, on a white background.

Immediately below the face, there was a list of words (neutrality, happiness, surprise, fear, anger, sadness, disgust), from which they had to select the emotion that they deemed the most appropriate to characterize the face. Next, participants judged how clearly they considered the expression recognizable on a 3-point Likert scale (ranging from “1 = not much” to “3 = very much”). The original wording was in Italian. The emotion was scored 0 if a different incorrect expression was selected. 5 s were allowed for perceiving and responding to the two queries. Participants were instructed to observe one face at a time and to respond within 5 s, not missing any answer. Only one choice per face was allowed. The task lasted about 15 min.

### Data Analysis

The individual scores obtained from each individual, for each of the 7 facial expressions and condition, underwent a 3-ways repeated-measures ANOVA whose factors of variabilities were: one between-groups named “sex” (with 2 levels, female and male), and two within-groups named “condition” (with 2 levels, natural and masked) and “emotion” (with 7 levels, happiness, neutrality, surprise, anger, sadness, fear, disgust).

In order to properly assess the statistical effect of the sex of participants (who were females in majority) in a balanced population, two subgroups of participants were created: the group of males comprised all male participants recruited (*N* = 47) and a blind selection of females (*N* = 47) chose on the basis of their date of birth (by paring each of the male with a same-age female). The statistical power achieved by the current sample size (*N* = 94) was computed using the program G*Power 3.1 (Faul et al., 2009) for comparing 2 independent groups.

As a result of this blind procedure, the age of the two sub-groups was identical (males: 23.042, fameless: 23.042). A 3-ways repeated-measures ANOVA was also performed on the data relative to this sample. Factors of variabilities were: one between-groups named “sex” (with 2 levels, female and male), and two within-groups named “condition” (with 2 levels, natural and masked) and “emotion” (with 7 levels, happiness, neutrality, surprise, anger, sadness, fear, disgust). Multiple *post hoc* comparisons were performed using Tukey’s test. Greenhouse-Geisser correction was applied in case of epsilon < 1 and epsilon corrected *p* value were computed.

## Results

**FIGURE 2:**
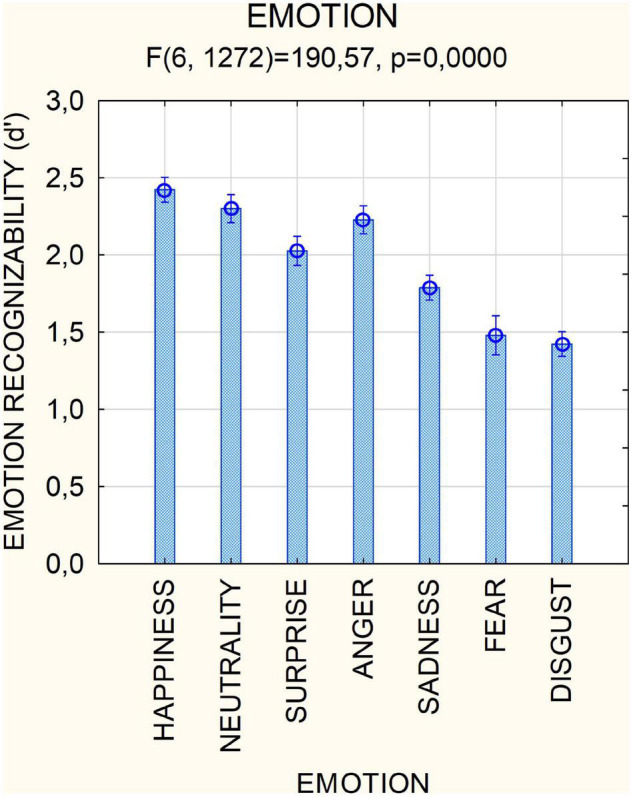
Mean scores of recognizability (along with SE values) attributed by participants (*N* = 214) to the various facial expressions regardless of masking condition. Scale ranged from 0 = “not much recognizable” to 3 = “very well recognizable”.

The factor condition was statistically significant [*F*(1,212) = 212;*p* < 0.001, ε = 1], with emotion recognizability being higher in the natural [2.31, standard error (SE) = 0.02] than masked (1.59, SE = 0.02) condition. The factor emotion was also significant [*F*(6,1272) = 191; *p* < 0.001, ε = 0.79, ε-corrected *p* value = 0.001]. *Post hoc* comparisons showed that overall positive emotions were recognized more easily than negative emotions (*p* < 0.001), except for anger, as shown in Figure 2 (neutral = 2.422, SE = 0.029; happy = 2.3, SE = 0.03; surprise = 2.02; SE = 0.03; anger = 2.22, SE = 0.03; sadness = 1.788, SE = 0.02; fear = 1.48; SE = 0.04; disgust = 1.42, SE = 0.02.). Happiness was recognized more easily (*p* < 0.001), the recognizability of fear and disgust was equally poor, while that of neutral and angry expressions was equally high.

**FIGURE 3:**
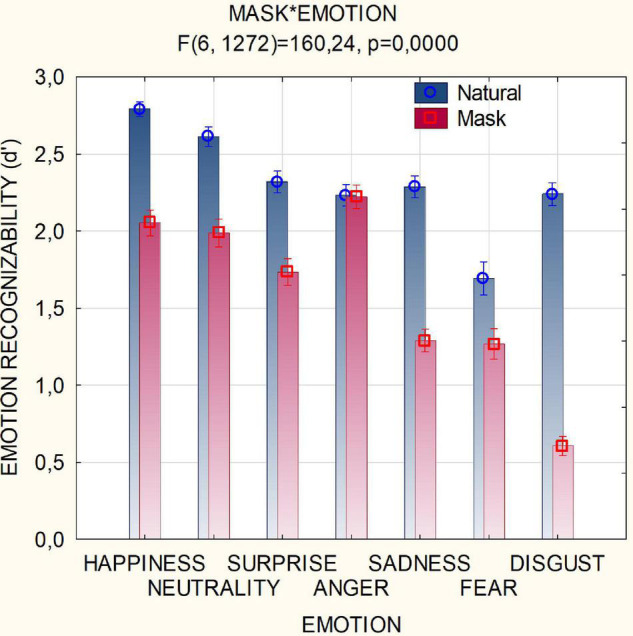
Mean scores of recognizability (along with SE values) attributed by participants (*N* = 214) to the various facial expressions as a function of masking condition. Scale ranged from 0 = “not much recognizable” to 3 = “very well recognizable”.

Surgical masks (covering the nose and mouth area) strongly reduced recognizability of all emotions, as shown by the statistical significance of condition × emotion [*F*(6,1272) = 160; *p* < 0.001, ε = 0.911, ε-corrected *p* value = 0.001], except for anger. *Post hoc* comparisons showed that neutral and happy expressions were equally well recognizable under the mask, but worse than angry expressions. Again, negative emotions such as disgust, sadness and fear were much poorly recognized than positive emotions in masked conditions. Figure 3 shows the mean scores for each facial expression as a function of the masking condition. Negative emotions such as sadness and disgust, more relying on the nose and mouth area expressivity, were most penalized by mask covering.

The sex of viewer affected the ability to recognize the emotions regardless of face covering, as shown by the significance of emotion × sex interaction [*F*(6,1272) = 4.14; *p* < 0.001, ε = 0.776, ε-corrected *p* value = 0.001]. The ANOVA performed on the two subgroups of 47 males and 47 females yielded the same significances as the main ANOVA, i.e.: condition (*p* < 0.001), emotion (*p* < 0.001), emotion × condition (*p* < 0.001) and emotion × sex interaction [*F*(6,552) = 4.138; *p* < 0.001, ε = 0.778, ε-corrected *p* value = 0.001].

**FIGURE 4:**
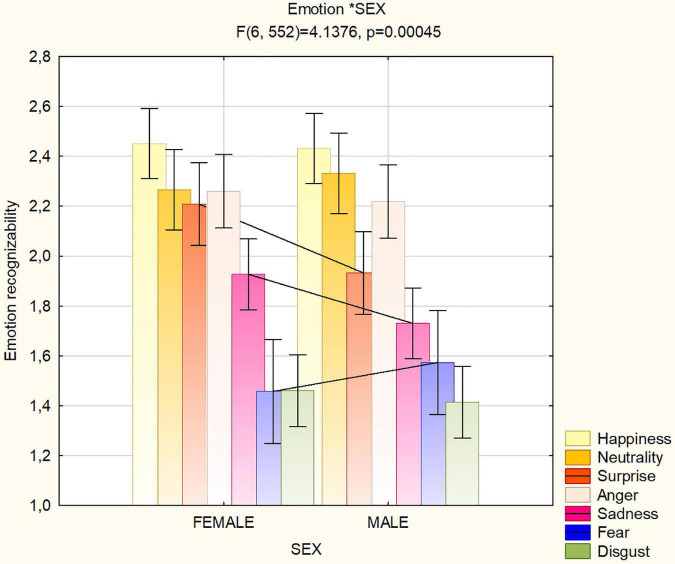
Mean scores of recognizability (along with SE values) attributed by female and male participants (*N* = 94) to the various facial expressions as a function of masking condition.

**FIGURE 5:**
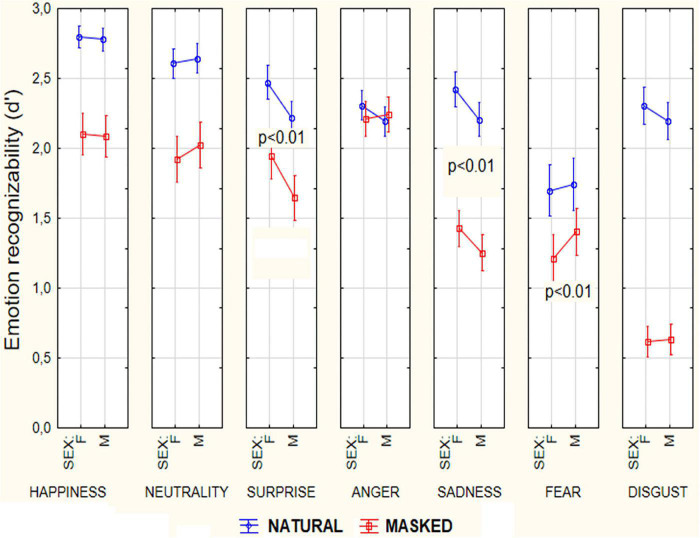
Mean scores (along with SE values) of recognizability attributed by participants (*N* = 94) to the various facial expressions as a function of sex of viewers and masking condition. Scale ranged from 0 = “not much recognizable” to 3 = “very well recognizable”.

As for the last interaction and similarly to ANOVA applied to the whole population (see Figure 4 for mean values and SEs), *post hoc* showed that while women were better at recognizing surprise (*p* < 0.004) and sadness (*p* < 0.05), males were better at recognizing fear expressions (*p* < 0.005). Simple effect analysis showed that this male advantage in recognizing fear was even stronger (see Figure 5) in the masked conditions (*p* < 0.004).

## Discussion

In the natural (mask less) conditions, positive emotions (happiness, neutrality, positive surprise) were recognized more accurately than negative emotions such as fear, sadness or disgust. This positive/negative valence distinction is based on the dichotomy on approach/avoidance attitude to emotions supported by previous neuroimaging and electrophysiological literature (Davidson, 1995; Balconi et al., 2017). Overall, masking heavily affected emotion comprehension with a 31% decay in recognizability (namely, going from 2.31 in the natural condition to 1.59 in the masked condition, on a scale where 0 indicated “not much recognizable” and 3 stood for “very well recognizable”). Overall, these findings fit with previous recent literature showing how facemasks reduce emotion recognition accuracy (Roberson et al., 2012; Carbon, 2020; Ruba and Pollak, 2020; Calbi et al., 2021; Marini et al., 2021; Grundmann et al., 2021; Carbon et al., 2022). In our study, face masking was most detrimental for sadness and especially disgust detection, than positive emotions such as happiness. This pattern of results agrees with previous studies, for example Marini et al. (2021), finding that sadness was the most affected and happiness the least affected expression by face masking.

However, we found that mask covering did not affect the recognition of angry faces, which replicates some findings obtained with non-digital masks by Noyes et al. (2021) (see their **Figure 7**), who also found that the mask and sunglasses conditions did not significantly differ in the angry expressions. The primacy of anger among the biologically relevant emotions has been shown by several studies (e.g., Mancini et al., 2020).

Conversely, the emotional display whose recognition was most affected by mask covering was disgust (also in Noyes et al.’s, 2021 study). Indeed, disgust’s more evident markers (nasiolabial lifting and grimacing and nose wrinkling) are hidden by surgical masks in the masking condition. At this regard it is known that successful recognition of anger versus disgust requires one to process information located in the eye/brow region (which was disclosed) as opposed to the mouth/nose region (which was covered by masks), respectively (Yitzhak et al., 2020). Again, in a study by Ponari et al. (2012) where emotion recognition was hampered by stimuli in which an upper or lower half-face showing an emotional expression was combined with a neutral half-face it was shown that neutral lower half-face interfered with recognition of disgust, whereas the neutral upper half (i.e., the eyes area) impaired the recognition of anger. This difference may probably explain the supremacy of anger and the poor recognition of disgust in the present study.

### Women Better at Recognizing Sadness and Surprise

In our study, females outperformed males in the recognition of sadness and surprise. Several evidences in the literature consistently reported a similar pattern of results for both sadness (Montagne et al., 2005; Deng et al., 2016; Li et al., 2020) and surprise (Montagne et al., 2005). In addition, according to some investigations, women seem to be more sensitive to sadness whereas men seem to be more sensitive to anger (Brody et al., 1995; Deng et al., 2016; He et al., 2018). In another study by Montagne et al. (2005) women were reported to be significantly more accurate than men at identifying sadness and surprise. Furthermore, Li et al. (2020)’s study, performed in 1,063 participants varying in sex and age, reported that women performed significantly better at recognizing facial expressions of sadness and disgust.

As for the specific effect of masking, Grundmann et al. (2021) tested 191 participants (52.9% female) aging from 19 to 79 years and found that emotion-recognition accuracy declined for masked (vs. unmasked) faces. More interestingly, they showed lower accuracy to being male vs. female, being old (vs. young), and to seeing an old (vs. young) target face. In a study by Calbi et al. (2021) involving only three affective displays (neutrality, happiness and anger) it was found that female participants gave more negative ratings than male ones when evaluating angry and neutral facial expressions, and more positive ratings when evaluating happy facial expressions. This was discussed in terms of women’ stronger sensibility to face expressivity and better decoding of emotions through facial expressions (e.g., McClure, 2000; Proverbio et al., 2007; Hall et al., 2010; Proverbio, 2021). Consistently, Hoffmann et al. (2010) found that women were better at identifying subtle, less intense emotions (such as sadness), but equally good at identifying clearly expressed emotions (such as fear). Apart from that, it is generally believed that women are more sensitive to emotional facial cues (Proverbio, 2017).

### Men Better at Detecting Fear

In this study, males outperformed women in recognizing fearful expressions (especially masked ones). The increased male ability to recognize fear (relying mostly on the processing of the eyes area, with the typical sclera enlargement) when faces were covered by surgical masks, might depend on the fact the eyes were even more focally attended in the masked condition, being the only uncovered face area. However, Sullivan et al. (2017), investigating the percentage of time young women and men spent fixating the eyes and mouth areas of facial expressions (including fear), found that both sexes spent 63.6% of their time looking at the eyes (and 36.4% of the time at the mouth) with no difference across sexes.

In the literature, a male advantage in the processing of fearful expressions is not commonly found, except for an fMRI study, observing regional brain responses to face versus shape identification, in which men showed more significant modulations by both fear and anger affective traits than women (Li et al., 2020).

On a different verge, Riva et al. (2011) have instead found that the observers’ ability to detect pain in a female face was lower than their ability to detect pain in male faces, i.e., that male pain faces are more easily processed at the reflexive level. Relatedly, Simon et al. (2006) in an fMRI study found that observing male (vs. female) individuals expressing pain activated in the observers a much greater threat-related response, including the activation of the ventromedial prefrontal cortex, posterior and anterior insula, somatosensory areas, and amygdala. In another study, where healthy subjects were provoked by money taken by an opponent and given the opportunity to retaliate, men showed a higher amygdala activation during provocation, and the amygdala activation correlated with trait anger scores in men, but not in women (Repple et al., 2018). As well-known amygdala nuclei are the brain structures most involved in fear and threat processing (Adolphs et al., 1995).

### Summary

Overall, while face masking reduced the comprehension of all facial expressions but anger (conveying an aggressive display), it was most detrimental for sadness and especially disgust detection (conveying a second person, more passive negative state). The larger impairment for the recognition of the above expressions might depend on their mainly relying on the expressivity of mouth (especially sadness: Smith et al., 2005; Wegrzyn et al., 2017) and nose areas (especially disgust: Yitzhak et al., 2020; Noyes et al., 2021), which were covered by masks. Instead, the angry expression was totally unaffected by face masking. This effect, different from previous studies, might be related to the ecological use of real and non-digital masks, allowing a more complex analysis of facial patterns.

In general, women showed a better performance for positive emotions, both in masked and natural conditions, and men for fear recognition (in natural but especially masked conditions). At this regard, it might be interesting to consider that sex differences in the hemispheric activation for emotion processing were reported. Cahill et al. (2001) found that enhanced memory for emotional video clips was associated with activity of the right amygdala in men, and of the left amygdala in women. In addition, an fMRI study investigating the emotional response to odors by Royet et al. (2003) found a sex difference in the activation of the left orbitofrontal cortex, which was greater in women compared to men. On the other side, Bourne and Watling (2015) found that for males, but not females, greater reported use of negative emotion strategies was associated with stronger right hemisphere lateralization for processing negative emotions. In the light of the well know right/left asymmetry for negative/positive emotions (Canli et al., 1998; Davidson et al., 1999) these studies might provide the neural underpinnings for the higher male accuracy in fear recognition (right amygdala), and of the higher female accuracy for detecting subtle positive emotional cues (e.g., Calbi et al., 2021), but further investigations are certainly needed to reach a definitive conclusion.

More in general, our study suggests the opportunity of studying the effect of face masking with really worn facemasks (instead of digitally applied ones) because there might be a difference in the way masks elastically respond to underneath facial muscles contractions, by deforming and stretching differently as a function of the facial expression. Furthermore, the typical inhalation associated, for example, to the surprised or fearful reaction (startle response), which results in mask sucking, will not be observable with digitally applied masks.

In general, wearing masks hampers facial affect recognition, and it might be particularly challenging for individuals with neuropsychiatric or neurodevelopmental conditions (Pavlova and Sokolov, 2021).

In this study, face masking was strongly detrimental to the comprehension of emotional markers, especially of non-aggressive negative states (such as sadness, disgust and fear). The only expression, whose recognition was not impaired by masking was indeed anger (associated with angry eyes, forehead wrinkling and contraction of mouth and lip muscles).

The primacy of anger among other more subtle emotions (such as sadness) has been reported in previous other studies (Öhman, 1993; Esteves et al., 1994; Fox et al., 2000), who found increased psychophysiological responding to masked angry faces relative to masked happy faces. The present data showed how face masking was able to polarize emotion comprehension toward the negative/positive opposite dimensions (happiness/anger or approach/withdrawal), while causing a deficit in social interaction and communication of softer emotions that usually trigger an empathic resonance in the observer (sadness, fear, disgust). The limited recognition of distressed people’s emotions might supposedly bring to a reduction of personal concern and empathic response (Israelashvili et al., 2020), within the population. This hypothesis strongly agrees with the recent findings by Rymarczyk et al. (2019), which, in a study using simultaneously recorded electromyography (EMG) and fMRI signals, showed that the perception of fear and disgust strongly activated brain regions involved in simulative processes and in empathy, such as mirror neurons (the fronto/parietal MNS) and limbic regions (e.g., the Anterior Insula (AI). Furthermore, the more empathic were the observers, the stronger was the reaction to these facial expressions. This seriously raises the question of a possible reduction in the observers’ empathic capacity in the absence of subtle, lower facial cues covered by facemasks. In fact, the present pattern of results indicates a selective decrease in the ability to recognize emotions that normally stimulate an empathic response (e.g., sadness, disgust, and fear) in face masking conditions.

### Study Limits

One possible limitation of this study is that static faces were used instead of dynamic videos for conveying affective information, since, naturally, the emotional valence of such stimuli is enhanced in naturalistic conditions (e.g., Ambadar et al., 2005; Rymarczyk et al., 2019). However, this study, and its novel pattern of results, should be compared with the pre-existing literature where masked static faces were used (Carbon, 2020; Ruba and Pollak, 2020; Calbi et al., 2021; Grundmann et al., 2021; Marini et al., 2021). It would be very interesting, in the near future, to investigate if this sparing of anger from the detrimental effects of masking can also be observed in dynamic conditions.

## Data Availability Statement

The original contributions presented in this study are included in the article/supplementary material, further inquiries can be directed to the corresponding author.

## Ethics Statement

The studies involving human participants were reviewed and approved by Ethical Committee of University of Milano-Bicocca. The patients/participants provided their written informed consent to participate in this study. Written informed consent was obtained from the individual(s) for the publication of any potentially identifiable images or data included in this article.

## Author Contributions

AP conceived and planned the experiment, performed statistical analyses and data illustration, and wrote the manuscript. AC prepared the stimuli and carried out the data collection. AP and AC interpreted the data. Both authors provided critical feedback and helped shape the research, analysis and manuscript.

## Conflict of Interest

The authors declare that the research was conducted in the absence of any commercial or financial relationships that could be construed as a potential conflict of interest.

## Publisher’s Note

All claims expressed in this article are solely those of the authors and do not necessarily represent those of their affiliated organizations, or those of the publisher, the editors and the reviewers. Any product that may be evaluated in this article, or claim that may be made by its manufacturer, is not guaranteed or endorsed by the publisher.
